# Hemophagocytic Lymphohistiocytosis Associated With Polyserositis and Salmonella typhi Infection

**DOI:** 10.7759/cureus.41182

**Published:** 2023-06-30

**Authors:** Humaira Achakzai, Gul Ghutai, Daud Khalil, Qazi Kamran Amin, Waqar Ullah

**Affiliations:** 1 Internal Medicine, Rehman Medical Institute, Peshawar, PAK; 2 General Medicine, Rehman Medical Institute, Peshawar, PAK

**Keywords:** hemophagocytes, megakaryocytes, salmonella typhi, polyserositis, hemophagocytic lymphohistiocytosis (hlh)

## Abstract

Hemophagocytic lymphohistiocytosis (HLH) is a rare but potentially fatal disease characterized by excessive immune response activation. Numerous conditions, including infectious etiologies, are implicated in its development. We report the case of a 16-year-old girl with HLH associated with polyserositis and *Salmonella typhi* infection. A 16-year-old girl presented with a high-grade fever and abdominal pain that had been ongoing for 20 days. She had been treated for malaria at a local hospital but was referred to our hospital due to the worsening of her condition. On examination, she was found to have an enlarged liver and spleen, pale skin, and hypotension, with bilateral basal crackles on chest examination. Her blood profile revealed pancytopenia, elevated C-reactive protein, and a deranged coagulation profile. Peripheral smears showed anisocytosis, microcytes, hypochromia in RBCs, and a few platelet clumps. A bone marrow biopsy revealed increased megakaryocytes and hemophagocytes. Ultrasound and computed tomography of the abdomen and pelvis showed hepatosplenomegaly, pericholecystic edema, mild ascites, and long-segment diffuse colonic wall thickening, suggesting pancolitis. Blood culture revealed *S. typhi*, which is rarely associated with HLH. The patient was started on the HLH-2004 protocol and showed improvement on the fourth day of initiating therapy, but due to a delayed diagnosis, the patient collapsed on the sixth day of admission.

HLH is a rare but life-threatening disease with various underlying causes. The diagnosis of HLH is challenging, and early diagnosis and prompt treatment are crucial for a better prognosis. The association between HLH and *S. typhi* infection is rare, and this case highlights the importance of considering unusual etiologies in HLH. Clinicians should be vigilant about this association, especially in endemic regions, to ensure early diagnosis and prompt treatment.

## Introduction

Hemophagocytic lymphohistiocytosis (HLH) is a rare but potentially fatal disease characterized by inappropriate and excessive immune response activation. The incidence of HLH is estimated to be 1 in 50,000 live births, with an equal distribution between infants and adults [1]. HLH results from the dysfunction of cytotoxic lymphocytes and natural killer (NK) cells, leading to macrophage hyperactivation, excess cytokine release, tissue damage, and multiorgan failure. Numerous conditions that trigger immune activation are implicated in the development of HLH, including infectious, autoimmune, metabolic, and neoplastic etiologies [2]. The clinical hallmarks of HLH include fever, cytopenia, splenomegaly, and systemic inflammatory response syndrome (SIRS)-like features, often including liver dysfunction. The HLH 2004 criteria are fever, splenomegaly, cytopenia affecting at least two of three lineages in peripheral blood, ferritin ≥500 μg/L, hypertriglyceridemia and/or hypofibrinogenemia, hemophagocytosis in bone marrow, spleen, or lymph nodes, low or absent NK cell activity, and a high level of soluble interleukin-2 receptor alpha chain (CD25) [3]. However, identifying the exact cause of HLH remains a challenge. In this case report, we present the case of a 16-year-old girl with HLH associated with polyserositis and *Salmonella typhi* infection.

## Case presentation

**Figure 1:**
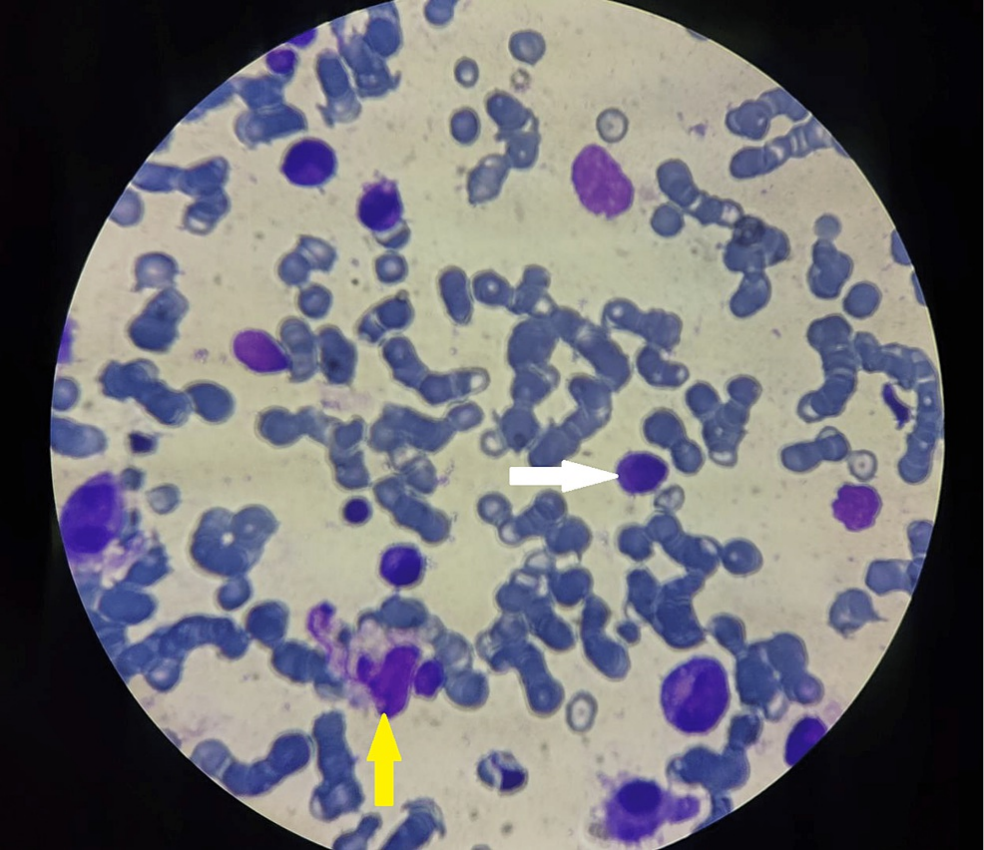
Bone marrow biopsy showing megakaryocytes (white arrow) and hemophagocytes (yellow arrow).

**Figure 2:**
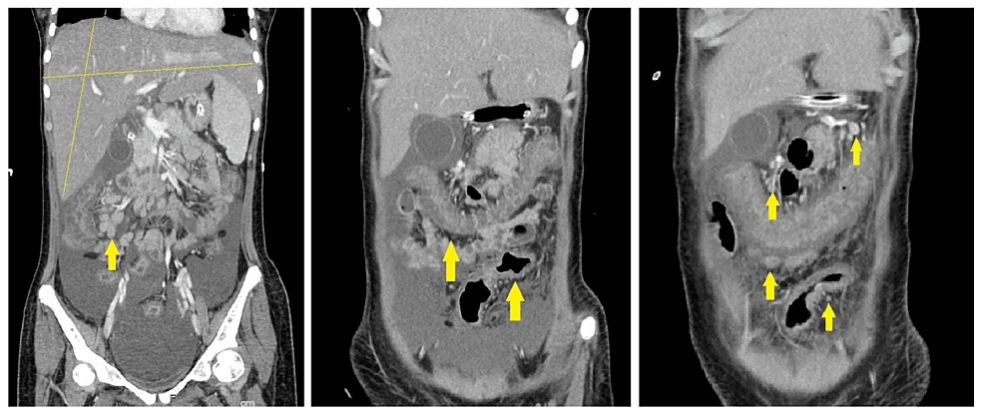
Multiple CT scan planes of abdomen and pelvis showing hepatosplenomegaly, moderate ascites, and multiple enlarged lymph nodes. CT: computed tomography.

A 16-year-old, unmarried Pakistani girl presented to us with chief complaints of high-grade fever and abdominal pain for 20 days. At first, she was treated for malaria in a local hospital, but due to the worsening of her condition, she was referred to our hospital, which is a tertiary care hospital in Peshawar City in the Khyber-Pakhtunkhwa province of Pakistan. On examination, she was toxic-looking, febrile, and hypotensive. She also had an enlarged liver and spleen. Her chest examination showed bilateral basal crackles. The patient was admitted to the medical ICU and started on broad-spectrum antibiotics, and supportive care was given. Investigations were carried out, which included a complete blood picture, viral profile, peripheral smear, liver and renal function tests, as well as bone marrow studies. The patient's blood picture showed pancytopenia with an initial RBC count of 2.98 (4.1-5.5 × 10^6^/μL), Hb of 8.1 (11-15 g/dL), WBC count of 0.51 (4-11 × 10^3^/μL), and platelet count of 130,000 (150,000-400,000 × 10^3^/μL). The CRP value was high at 36.16 (0-0.5%), serum ferritin was >1675.56 (14-150 ng/ml), and the coagulation profile was deranged with an INR of 1.3 (0.9-1.3), APTT of 48 (28 seconds), and low serum fibrinogen levels of 192 (200-400 mg/dl). The patient's viral profile was negative for Hep B (HbsAg), Hep C (anti-HCV antibodies), and HIV. The malarial parasite, dengue serology, and antinuclear antibodies were all negative (Table 1). Peripheral smear revealed anisocytosis, microcytes, hypochromia in RBCs, anisocytosis, megaplatelets, and a few platelet clumps in platelets, while WBCs showed normal morphology (Figure 1). Ultrasound and computed tomography scans of the abdomen and pelvis were also done, which showed hepatosplenomegaly, pericholecystic edema with sludge, mild ascites, long-segment diffuse colonic wall thickening suggesting pancolitis with prominent vasa recta, multiple prominent lymph nodes along the right colon, multiple sub-centimeter mesenteric and para-aortic lymph nodes, and moderate ascites (Figure 2). A surgical opinion was also taken for a lymph node biopsy to confirm any suspected lymphoma/cancer; however, they advised conservative treatment as they could not proceed due to her deranged coagulation profile. Her chest X-ray showed moderate pleural effusion, which worsened every day, even though the patient did not have any suggestive clinical signs. Further confirmatory tests, such as fluid analysis, could not be done due to her increased risk of bleeding. The patient's blood culture revealed *S. typhi*. Bone marrow biopsy results showed increased megakaryocytes and hemophagocytes (+++), with no extramedullary cells seen, which directed our diagnosis toward HLH as she fulfilled five out of eight HLH-2004 criteria.

**Table 1:** Shows the daily trend of biomarkers and other investigations carried out during the admission time period. RBC: red blood cells, Hb: hemoglobin, WBCs: white blood cells, INR: International normalization ratio, PT: prothrombin time, APTT: activated partial thromboplastin time, ALT: alanine transaminase, ALP: alkaline phosphatase, LDH: lactate dehydrogenase, HbsAg: hepatitis B, HIV: human immune-deficiency virus, EBV: Epstein-Barr virus, ANA: anti-nuclear antibodies.

Investigations	Day 1	Day 2	Day 3	Day 4	Day 5	Normal range
RBCs	2.98	2.98	3.5	4.4	4.05	4.1–5.5 (× 10^6^/μL)
Hb	7.6	8.1	10	11.8	13	11–15 (g/dL)
WBCs	0.76	0.51	1.0	1.13	4.34	4–11 (× 10^3/μL)
Platelets	114	128	170	170	296	150–400(× 10^3/μL)
Neutrophil	15.8	15.7	28.3	44.2	48.2	40–75%
Monocytes	26.3	21.6	17	13	15.7	2–10%
Lymphocytes	0.44	0.32	0.5	0.4	1.12	1.5–4%
C-reactive protein	36.16					0–0.5%
Serum ferritin	>1675.56					14–150 (ng/ml)
INR	1.3	1.9	1.8	2.7	2.3	0.9–1.3
PT	14.3	21.1	20.1	23.1	24.8	10–13 seconds
APTT	48	45.1	43	41.8	40	21–35 seconds
Serum fibrinogen	192					200–400 (mg/dL)
ALT	95			198	135	5–35 (U/L)
Total bilirubin	1.11			3.39	4.21	0.2–1.1 (mg/dL)
Serum albumin	2.1			3.1	3.4	3.2–4.5 (g/dL)
ALP	146			140	143	51–332 (U/L)
LDH	826					135–214 (U/L)
Serum creatinine	0.69		0.69	0.43	1.32	0.59–0.86 (mg/dL)
Serum urea	10			20	20	10–50 (mg/dL)
HbsAg	Negative					
Hepatitis C (anti HCV antibodies)	Negative					
HIV	Negative					
Malarial parasite	Negative					
Dengue serology	Negative					
EBV	Negative					
Blood group	B positive					
ANA profile	Negative					

The patient was started on the HLH2004 protocol, which consists of an eight-week induction therapy followed by a maintenance phase. The induction phase included dexamethasone (10 mg/m²/day) and etoposide (150 mg/m²/day), which should be continued for eight weeks. A fresh frozen plasma of 150 ml was transfused, and injections of furosemide (20 mg intravenous) were given twice daily to improve her pleural effusion and ascites. The patient, although critically ill, started showing improvement on the fourth day of initiating therapy, with an improving blood count and coagulation profile, as well as a decrease in ascites and pleural effusion, and an increase in serum albumin levels of 2.1 to 3.4 (3.2-4.5 g/dl). However, the patient's other biochemical markers were still deranged, and on an increasing trend, ALT levels were elevated at 95 (5-35 U/L) and total bilirubin was 1.11 (0.2-1.1 mg/dl) initially, which increased to 4.21. Serum creatinine value of 0.69 (0.59-0.86) initially, which increased to 1.32, and a serum urea value of 10 (10-50 md/dl), which increased to 20. On the sixth day, the patient went into sudden cardiopulmonary arrest. The patient was unresponsive, and her pulse was not palpable. Her blood pressure dropped to 60/40 mmHg, and her oxygen saturation decreased to 60%. An emergency code was activated, and the patient was immediately intubated and ventilated with high oxygen flow. Intravenous fluids, adrenaline, and norepinephrine were given to maintain her blood pressure. Despite all efforts, the patient could not be revived.

## Discussion

*Salmonella typhi* infection is a rare trigger for HLH, and this case report highlights the importance of considering HLH in patients with *S. typhi* infection presenting with clinical features suggestive of HLH [4]. Early recognition and prompt initiation of therapy are essential for improved outcomes in such cases. This case report also highlights the effectiveness of etoposide and dexamethasone in HLH treatment, consistent with previous reports [5]. As per the HLH-2004 diagnostic criteria, HLH is diagnosed when at least five of the eight criteria listed are fulfilled. These criteria are fever, splenomegaly, cytopenia affecting at least two of three lineages in peripheral blood, ferritin ≥500 μg/L, hypertriglyceridemia and/or hypofibrinogenemia, hemophagocytosis in bone marrow, spleen, or lymph nodes, low or absent NK cell activity, and a high level of soluble interleukin-2 receptor alpha chain (CD25). Recent studies have suggested the potential use of targeted therapy for HLH based on genetic and immune markers. Genetic analysis may also help identify familial HLH cases, which require long-term monitoring and management. However, the high cost and limited availability of genetic testing remain challenges in low-resource settings [6,7].

The patient in this case report was treated with a combination of dexamethasone and etoposide as per the HLH-2004 protocol. Dexamethasone was given at a dosage of 10 mg/m^2^/day (maximum dose 20 mg); this should be continued for the first two weeks, followed by 5 mg/m^2^/day (maximum dose 10 mg) for the next two weeks. Etoposide was given at a dosage of 150 mg/m^2^/day for three days, which is given for the first two weeks and then weekly for the next two weeks. The patient showed improvement with this treatment regimen. Other treatments such as cyclosporine, intravenous immunoglobulin, and antithymocyte globulin are used in refractory cases [8].

Several recent studies have reported the successful use of the HLH-2004 protocol in pediatric patients with HLH. A study by Henter et al. reported that the HLH-2004 protocol is the most effective treatment regime for HLH [6]. Another study by Wang and Yang found that the HLH-2004 protocol was effective in treating HLH associated with Epstein-Barr virus infection [9]. These studies highlight the importance of early recognition and prompt treatment of HLH using the HLH-2004 protocol.

This case report has several strengths. First, it highlights the association of HLH with *S. typhi* infection, which is a relatively uncommon presentation. Second, the case report demonstrates the efficacy of etoposide and dexamethasone in the management of HLH, consistent with previous studies. Third, the report emphasizes the importance of early recognition and prompt treatment in HLH, leading to a good clinical outcome. It adds to the limited literature available on this topic and emphasizes the need for prompt recognition and appropriate management of HLH in patients with *S. typhi* infection.

The limitations of this case report include the lack of genetic testing, which could have aided in identifying the underlying genetic predisposition to HLH, and the limited follow-up data.

## Conclusions

HLH is a rare but potentially fatal disease that requires prompt recognition and appropriate management using the HLH-2004 protocol. This case report highlights the association of HLH with *S. typhi* infection and emphasizes the importance of considering HLH in patients presenting with systemic inflammatory response syndrome-like features. Clinicians should be aware of this association and initiate early treatment to improve patient outcomes. It also suggests the effectiveness of etoposide and dexamethasone in HLH management. Further studies are required to assess the use of targeted therapy in HLH and identify genetic predispositions in patients with HLH.
